# Early Supplementation with Starter Can Improve Production Performance of Lambs but this Growth Advantage Disappears after 154 Days of Age

**DOI:** 10.3390/ani13030372

**Published:** 2023-01-21

**Authors:** Jianfeng Xu, Fadi Li, Zhendong Zhang, Chen Zheng, Zhenfeng Shen, Zhiyuan Ma, Jing Wang, Fan Zhang, Jiaqi Wang, Hang Ran, Ying Yun, Ting Liu

**Affiliations:** 1 College of Animal Science and Technology, Gansu Agricultural University, Lanzhou 730070, China; 2 College of Pastoral Agriculture Science and Technology, Lanzhou University, Lanzhou 730020, China

**Keywords:** *Hu* lambs, starter, early supplementation, growth performance, slaughter performance

## Abstract

**Simple Summary:**

Early nutritional intervention in young animals can regulate physiological and growth performance at later stages. Previous studies have shown that early supplementation with starters can improve lamb growth performance; however, most of them focus on physiological changes in the short period before and after weaning of lambs, while reports on sustained effects on growth performance in later stages of lambs are scarce. This study investigated the sustained effects of early starter supplementation on the performance of *Hu* lambs during the pre-weaning, post-weaning, and fattening periods.

**Abstract:**

The aim of this experiment was to study the sustained effects of early supplementation with a starter on the performance of lambs during the pre-weaning, post-weaning, and fattening periods. Sixty male *Hu* lambs (3.59 ± 0.05 kg) were randomly assigned to two (30 lambs per group) treatments. The lambs were fed milk replacer from three days of age. The early supplementation (ES) group was supplemented with a starter ration at seven days of age, the control (CON) group was supplemented at 21 days of age, and lambs in both groups were weaned from milk replacer at 28 days of age. Eight lambs from each group were randomly slaughtered at 98 days of age, and the remaining lambs were fed the same nutrient level of a fattening ration until slaughter at 200 days of age. The results showed that early supplementation with starter significantly improved average daily feed intake (ADFI) and average daily weight gain (ADG) in the pre-weaning period (7–28 days of age), and ADFI and slaughter performance (live weight before slaughter, carcass weight and dressing percentage) in the post-weaning period (29–98 days of age, *p* < 0.05). In addition, early supplementation with the starter had no significant effect (*p* > 0.05) on ADFI, ADG, and slaughter performance, but significantly decreased (*p* < 0.05) the feed-to-gain ratio (F/G) of lambs during the fattening period (99–200 days of age). In addition, early supplementation with starter increased the ratio of rumen and reticulum weight to total stomach weight in lambs (*p* < 0.05). In conclusion, early supplementation with starter can reduce feed costs and improve the performance of lambs, while the growth advantage produced by early supplementation had initially disappeared by 154 days of age.

## 1. Introduction

Early weaning of lambs is an important strategy for shortening the production cycle of ewes, increasing the frequency of lambing, and improving the economic efficiency of housing farming [1,2]. Compared to natural weaning, early weaning has more significant effects on various physiological functions in young ruminants [3,4]. Studies have shown that early weaning can enable the digestive system of young ruminants to be exercised early and improve growth performance and slaughter rates [5,6]. However, a challenge for early weaning is weaning stress, which manifests itself in the occurrence of physiological reactions such as decreased feed intake and body weight, and increased diarrhea [7,8,9,10]. Studies have indicated that supplemental feeding with an appropriate amount of solid starter can reduce the stress caused by early weaning in lambs [11]. Early supplementation with a starter is an effective way to achieve early weaning, promote early rumen development, and ensure healthy growth of lambs [12]. Another study confirmed that supplementing pre-weaning lambs with alfalfa starter increased average daily weight gain (ADG), body weight, feed intake, and carcass weight of lambs pre- and post-weaning [13]. Early feeding of the starter triggered metabolic imprinting in young ruminants, which resulted in a good growth rate and feasibility for improved production performance [14,15,16]. At present, many studies focus on the physiological changes of lambs in the short term during the pre- or post-weaning periods [17]; however, little attention has been paid to long-term studies on the growth period of lambs, which limits the application and development of starters in the production of lambs. We hypothesize that the growth advantages of early supplementation with starters will extend into the later part of the lamb’s growth period, and our purpose in implementing early supplementation in lambs is to maximize the breeding benefits by exploiting the potential of the entire growth period. Therefore, this experiment was designed to investigate the effects of early supplementation on the growth performance, slaughter performance, and gastrointestinal development of *Hu* lambs at different growth stages.

## 2. Materials and Methods

### 2.1. Experimental Design, Animal and Diets

The experiments were conducted at Zhongtian Sheep Farm in Minqin County, Gansu Province, P. R. China, from July to 2021 to January 2022. The experimental animals were obtained from Lanzhou New Area Tian Xin Sheep Industry Co (Lanzhou, China). 

Sixty male *Hu* lambs with similar genetic background and similar body weights (BW) (3.59 ± 0.05 kg) were randomly selected from newborn twin *Hu* lambs for this study. We used a paired trial design and the twin lambs were assigned to different treatments. The lambs were born within a week of each other. After birth, the lambs were fed colostrum until 3 days of age. Thereafter, a milk replacer containing milk components was fed at 2% of each lamb’s body weight, three times a day, at 8:00, 14:00, and 20:00. At 7 days of age, the lambs were randomly divided into two treatment groups, with 30 lambs in each group. The treatments included ES group (early supplementation group: added starter ration at 7 days of age, BW = 4.57 ± 0.80 kg) and CON group (control group: added starter at 21 days of age, BW = 4.42 ± 0.60 kg). From 21 to 27 days of age, the amount of milk replacement was gradually reduced by the same amounts for both groups until both groups of lambs were completely weaned at 28 days of age. Both groups continued to be offered starter twice daily (8:00 a.m. and 19:00 p.m.) until 98 days of age, when eight lambs from each group in different locations of the sheep house were randomly selected and slaughtered for sampling. The remaining lambs were fed a fattening ration twice daily (8:00 a.m. and 19:00 p.m.) until 200 days of age (at this time, the feed intake and body weight of the lambs plateaued), and all remaining lambs were slaughtered for sampling. Each lamb had ad libitum access to food and water throughout the experiment.

The milk replacer contained ≥23% protein, ≥12% crude fat, and ≤3% crude fiber, and was produced by Beijing Precision Animal Nutrition Research Center Co. (Beijing, China). The starter and fattening rations used in the experiment were processed and produced by Gansu Runmu Biological Engineering Co. (Jinchang, China). The feed was prepared as a mixture of pellets approximately 3.5 mm in diameter and 1–2 cm in length. The proportion of ingredients formula for starter and fattening rations refer to the “Standard for Feeding Meat Sheep in the Agricultural Industry of the People’s Republic of China” (NY/T816-2004) [18]. The ingredients and nutritional composition of the starter and fattening rations are shown in Table 1.

*Hu* lambs were kept individually in metabolic cages (0.9 m × 1.2 m) until 28 days of age, and at 29 days of age, they were individually transferred to separate pens (1 m × 1.2 m). Each cage and pen was equipped with a trough and a drinking trough to ensure that they were fed and watered freely. All cages and pens were numbered with the lamb’s ear tag and located in a temperature-controlled (20.0 ± 0.5 °C) and ventilated sheep house. During the experimental period, all lambs were treated for internal and external parasites and immunized against according to the requirements of standardized immunization on sheep farms. Sheep houses were cleaned and disinfected weekly.

### 2.2. Methods of Sampling and Measurement

The starter residue of each lamb was weighed before feeding each morning to calculate feed intake. Daily feed intake and weekly body weight (the lambs were weighed each morning before feeding, 7:00 a.m.) were recorded. Lambs were fasted overnight prior to weekly weighing. ADG was calculated as the final minus initial body weight divided by the trial period. Body height, body slope length, chest circumference, and tube circumference of the lambs were measured with a tape measure before the weekly morning feeding. Body height was defined as the vertical distance from the highest point of the vertebral scapula to the ground. The body oblique length was defined as the distance from the shoulder to the end of the sciatic tuberosity. Chest circumference was defined as the length of one circumference around the chest from the posterior end of the scapula. The circumference of the canal is the circumference of the thinnest part of the canal bone, which is approximately one-third of the distance from the bottom of the left foreleg tibia. 

Every morning before feeding, feces excreted by each sheep within 24 h were collected in aluminum boxes and accurately weighed. Half the total weight of fecal sample was dried in an oven at 65 ºC, and the dried feces of lambs in each group were evenly mixed at the end of the digestive and metabolic experiments and stored at normal temperature for subsequent nutrient analysis. Feed and fecal samples were analyzed for dry matter DM (method 930.15) according to the AOAC method [19]. The neutral detergent fiber (NDF) and acid detergent fiber (ADF) contents in the samples were determined using the method described by Van Soest et al. [20].

After each stage of the experiment, the animals were weighed after overnight fasting (12 h) prior to being slaughtered. Slaughter was performed according to the standard issued by the Gansu Provincial Veterinary Bureau (Gansu Veterinary Medicine [2016] No. 69). After slaughter, the head and hooves were removed, and the heart, liver, spleen, lungs, kidneys, stomach, small intestine, and large intestine tracts were separated on a clean operating table. The surface fat and contents of the stomach and intestines were removed, rinsed with saline, dried with sterile gauze, and the weight of the tissues and organs and the length of each intestinal tract were measured. The weight of the experimental lambs weighed before slaughter was considered as the live weight before slaughter (LWBS), and after slaughter and bloodletting, the carcass was weighed by removing the head, hooves, skin, and viscera (retaining the kidney and perinephric fat). The slaughter rate was calculated as the ratio of carcass weight to the weight of the live carcass before slaughter.

### 2.3. Statistical Analyses

Data related to the ADG, average daily feed intake (ADFI) and feed-to-gain ratio (F/G) were analyzed with R statistics (v3.5.1), applying the following model:

$$Y_{\mathrm{ijk}}=\mu +T_{i}+A_{j}+{\left(T:A\right)}_{\mathrm{ij}}+\mu_{k}+\beta_{K}+\epsilon_{\mathrm{ijk}}$$

where $Y_{\mathrm{ijk}}$ is the value of lamb k measured at treatment i and age j, μ is the overall mean, $T_{i}$ is a fixed effect for two treatments (ES and CON, i = 1, 2), $A_{j}$ is a fixed effect for age for four stages (pre-weaning, post-weaning, fattening period and overall, j = 1, 2, 3, 4), (T: A)_ij_ is a fixed effect for the interaction of treatment and age, $\mu_{k}$ is a random for lambs of different weaning days of age), $\beta_{K}\;$is a covariate for the 7-day-old weight and $\epsilon_{\mathrm{ijk}}$ is the random residual error.

Data related to body weight, slaughter performance, and the weight of tissues and organs were subjected to an independent-samples *t*-test using SPSS (IBM Corp. released 2019 and IBM SPSS Statistics for Windows, Version 26.0. Armonk, NY, USA: IBM Corp.). The model used was as follows:

$$t=\frac{\bar{x_{1}}-\bar{x_{2}}}{S_{\bar{x_{1}}-\bar{x_{2}}}}\;$$

where $\bar{x_{1}}$ and $\bar{x_{2}}$ are the means of different treatments, and $S_{\bar{x_{1}}-\bar{x_{2}}}$ is the standard error of the mean difference. The significance level was set at *p* < 0.05. 

The ADFI and ADG of lambs at different ages were collated and bar graphs were plotted using Origin (2021; OriginLab, Hampton, NY, USA). The feed intake and body weight of each lamb were collated and plotted as a scatter plot using Origin 2021, and the Boltzmann function was used to fit a nonlinear growth curve to visualize the changes in feed intake and body weight of the two groups of lambs. The function model used was as follows:

$$y=\frac{A_{1}-A_{2}}{1+e^{\left(x-x_{0}\right)/dx}}+A_{2}$$

where $A_{1}$ is the initial value,$\;A_{2}$ is the final value, $x_{0}$ is the center value of age, dx is the time constant, and y is the feed intake or body weight of the experimental and control groups.

## 3. Results

### 3.1. Effect of Early Supplementation on Growth Performance of Hu lambs

**Figure 1:**
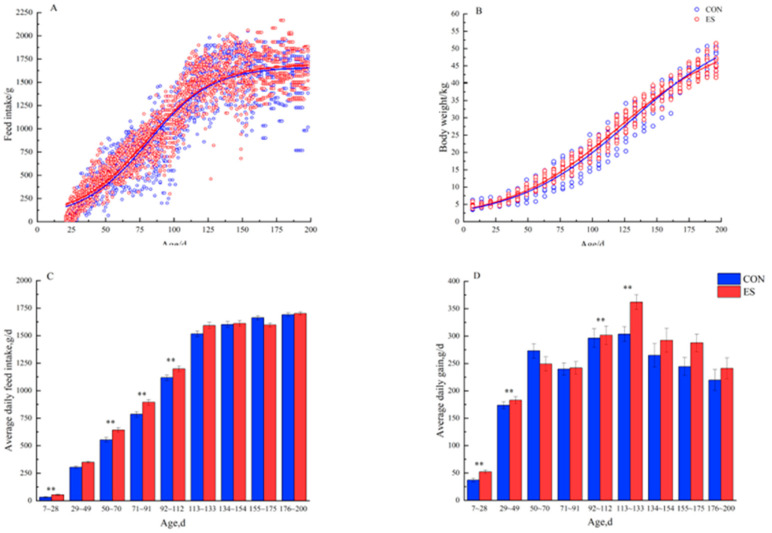
Growth performance of lambs at different ages (*n* = 22). Growth performance includes: (**A**) Feed intake, (**B**) body weight, (**C**) average daily feed intake, and (**D**) average daily gain. ** indicate significant differences between treatment groups.

The effect of early supplementation on the growth performance of lambs is shown in Figure 1 and Table 2. Scatter plots of body weight and feed intake throughout the growth period showed that feed intake and body weight were consistently higher in the early supplementation group but stabilized after 154 days of age in both groups of lambs. The ADG of the lambs were significantly affected (*p* < 0.05) by the treatment × age during the pre- and post-weaning periods. The ADG, ADFI and F/G of the lambs were significantly affected (*p* < 0.05) by the age during the pre-weaning, post-weaning, fattening period and overall. The ADG of lambs in the ES group was significantly higher than that of the CON group during the pre-weaning (7–28 days of age, *p* < 0.05), but there was no significant difference between the two treatments of lambs during the post-weaning (29–98 days of age), fattening period (99–200 days of age), and overall period (7–200 days of age, *p* > 0.05). Additionally, the ADG of lambs in the ES group was significantly higher than that in the CON group at 29–49 days of age, 92–112 days of age and 113–133 days of age (*p* < 0.05). The ADFI of lambs in the ES group was significantly higher than that in the CON group during the pre-weaning (7–28 days of age), post-weaning (29–98 days of age), and overall period (7–200 days of age, *p* < 0.05), but there was no significant difference between the two groups during the fattening period (99–200 days of age, *p* > 0.05). In addition, the ADFI of lambs in the ES group was significantly higher than that of the CON group at 50–70 days of age, 71–91 days of age and 92–112 days of age (*p* < 0.05). The F/G of lambs in the ES group was higher than that in the CON group during the pre-weaning period (7–28 days of age, *p* < 0.05), whereas no significant difference was observed between the two groups during the post-weaning period (29–98 days of age, *p* > 0.05). During the fattening period (99–200 days of age), the F/G of lambs in the ES group was lower than that in the CON group (*p* < 0.05).

The effect of early supplementation on the body size of *Hu* lambs is shown in Table 3. The body height of lambs in the ES group was significantly lower than that of lambs in the CON group (*p* < 0.05), and the body length was significantly higher than that of lambs in the CON group (*p >* 0.05) at 28 days of age. At 56 and 112 days of age, the body height of lambs in the ES group was significantly higher than that in the CON group (*p* < 0.05), and the tube circumference of lambs in the ES group was significantly higher than that in the CON group at 112 days of age (*p* < 0.05). At 140 days of age, the body length of lambs in the ES group was significantly greater than that in the CON group (*p* < 0.05).

### 3.2. Effect of Early Supplementation on Faecal Dry Matter and Fibre Digestibility of Hu lambs

The effects of early supplementation on faecal dry matter and fibre digestibility of *Hu* lambs are shown in Table 4. At 133–154 and 155–175 days of age, the digestibility of DM in lambs in the ES group was significantly higher than that in lambs in the CON group (*p* < 0.05). At 155–175 and 176–200 days of age, the digestibility of NDF and ADF in lambs in the ES group was significantly higher than that in lambs in the CON group (*p* < 0.05).

### 3.3. Effect of Early Supplementation on Slaughter Performance of Hu lambs

The effects of early supplementation on slaughter performance of *Hu* lambs are shown in Table 5. At 98 days of age, the live weight before slaughter and carcass weight of lambs in the ES group were significantly higher than those in the CON group (*p* < 0.05); however, at 200 days of age, there were no significant differences in the slaughter indices of lambs between the two groups (*p* > 0.05).

### 3.4. Effect of Early Supplementation on Gastrointestinal Tract Development of Hu lambs

The effects of early supplementation on the gastrointestinal index in 98- and 200-days-old *Hu* lambs are shown in Table 6. The weights of the rumen and reticulum, the percentage of reticulum to total compound stomach weight, and the weight of jejunum were significantly higher in the ES group than in the CON group at 90 days of age (*p* < 0.05). At 200 days of age, the percentage of rumen weight to pre-slaughter live weight, percentage of rumen weight to total compound stomach weight, and percentage of reticulum weight to total compound stomach weight were significantly higher in the ES group than in the CON group (*p* < 0.05), and the percentage of duodenal weight to total gastrointestinal tract was significantly lower in the ES group than in the CON group (*p* < 0.05). The effects of early supplementation on the intestinal length of *Hu* lambs are shown in Table 7. There was no significant difference in gut length or relative length between the two groups at 98 and 200 days of age (*p* > 0.05).

## 4. Discussion

In this experiment, early supplementation of lambs with starters increased the ADG of lambs at 7–28, 29–49, 92–112 and 113–133 days of age and increased ADFI before and after weaning. The growth performance of lambs is a key factor in measuring farming efficiency [21]. Even very small changes in nutritional supply during the early life of animals may lead to profound and lasting effects on the subsequent life of the organism, including changes in tissues and even the organism [22]. A similar study found that when 10-day-old lambs were supplemented with a starter containing alfalfa, the feed intake and nutrient intake of the lambs were improved, and the ADG and body weight were increased in the pre- and post-weaning periods [23]. After the lambs adapted to the new feeding method, the early weaned lambs grew faster than the normal-weaned lambs [24]. However, in our experiment, there was no significant difference in the ADG of lambs after weaning and fattening. Studies have shown that the rumen of lambs starts to develop from three weeks of age, when digestive and metabolic functions also start to develop [25]. In our experiment, the control group was supplemented with starter at 21 days of age to stimulate rumen development. The ADG of lambs in the early supplementation group was significantly higher than that of the control group at 28–49 days of age, whereas the daily feed intake was similar between the two groups. While the rumen development of the control group lambs was lower than that of the early supplemented lambs, the degree of rumen development in the control group lambs probably met the weaning requirements by 28 days of age; therefore, there was no difference in ADG between the two groups at post-weaning. For supplemental starter, the effect of different fiber sources on the rumen varies, which is highly dependent on the fiber content of the feed and the amount fed [26]. Most studies have shown that young ruminants supplemented with starters can improve production performance [27,28], but this advantage is not sustained throughout the lamb’s growth period. In our experiment, lambs in the early supplementation group gained significant weight at 92–112 days of age and 113–133 days of age, but this advantage faded later. Combining the scatter plots of body weight and feed intake throughout this growth period shows that the early supplementation group had consistently higher feed intake and increased nutrient intake, so that body weight was higher than that in the control group. After 154 days of age, the weight of lambs in both groups stabilized at a point in time when the growth advantage produced by early supplementation with starter diminished and disappeared. A study showed that the weight gain advantage of early weaning of lambs was reflected at 66–120 days of age [29], which differs from the results of the present experiment, probably because of the different weaning ages.

Body size is also an important index for evaluating the condition of animals. In our study, the body size of lambs in the early supplementation group grew faster during the fattening period, which also indicated that the body condition of lambs in the early supplementation group during the fattening period was better. Studies have shown no significant effect of early supplemental feeding age or physical form of the starter feed on the body size of ruminants [30,31]. However, one study also showed that the level of supplemental concentrate feeding affected the body condition of the lambs [32]. The early supplementation group ingested relatively more fattening rations during the fattening period, which may have been the cause of the effect on their body condition.

In production, the level of feed conversion ratio also affects the level of breeding efficiency, and a high feed conversion ratio (low feed to gain ratio) can save a lot of feed and reduce production costs. Feed intake and digestive absorption in relation to rumen development. The establishment of rumen function in lambs started at 3 weeks of age when the proliferation of rumen epithelial cells was sensitive to stimulation by vegetable proteins or solid feeds. We found that the feed conversion rate of lambs in the early supplementation group was lower in the pre-weaning period, which should be due to the fact that the rumen of lambs is not mature and has a lower conversion rate for solid feeds. Of course, the separation from the ewe and the stress reaction to solid feeds could also affect the lamb’s ability to digest solid feeds [33]. However, the feed conversion rate of lambs in the early supplementation group was higher during the fattening period, and early supplementation improved the performance of lambs during the fattening period. In this study, the dry matter digestibility of lambs in the early supplementation group was significantly higher than that of the control group at 133–154 and 155–175 days of age, with no significant difference at 175–200 days of age, which was consistent with the trend in the ADG of lambs at that stage. The digestibility of NDF and ADF was higher in the early supplementation group than in the control group during the fattening period, which was possibly due to the improvement in rumen function and the increase in the type and number of rumen microorganisms at this stage. Early supplementation with solid feeds increased rumen weight and volume and enhanced rumen metabolism of lambs [34,35].

Slaughter rate and carcass weight are important indicators of the slaughter performance of animals; the higher the slaughter rate, the greater the meat production capacity. In this experiment, the live weight before slaughter of the slaughtered lambs was based on the average weight, which can be considered a visual reflection of the effect of early supplementation on the post-weaning and fattening stages of the lambs. The study showed that early weaned lambs had higher pre-slaughter live weight and carcass weight than naturally weaned lambs at 90 days of age, but the difference in slaughter rate was not significant [36]. A similar study showed that early weaned lambs (weaned at 10, 20, and 30 days of age) had significantly higher pre-slaughter live weight and carcass weight than normal lambs in all groups at 90 days of age for slaughter, with no significant difference in slaughter rate [37]. From these results, it can be concluded that early weaning of lambs with milk replacers or solid starters can improve body quality and increase meat production, which is conducive to the production and breeding of lambs. Similarly, the results of our experiment indicated that at 98 days of age, the live weight before slaughter and carcass weight of lambs in the early supplementation group were significantly higher than those in the control group, but the difference in slaughter rate was not significant. Supplemental feeding to pasture lambs could also increase live weight before slaughter and carcass yield at three months of age [38]. Early supplementation not only increased the energy intake of the diet but also had a positive effect on rumen development, resulting in higher weight gain in supplemented lambs [11]. There was no significant difference in the live weight before slaughter and carcass weight of lambs in the early supplementation group compared with the control group when the fattening period ended at 200 days of age. During the fattening period, the early supplementation with starter-treated lambs was similar to the control lambs in terms of feed intake and weight gain, so there was no difference in slaughter indices, indicating that the growth advantage generated by early supplementation had disappeared at that time.

One of the reasons for early supplementation to promote growth may be its stimulating effect on rumen development. In this study, the rumen and reticulum weights and the percentage of reticulum to total compound stomach weight were significantly higher in the early supplementation group than in the control group at 98 days of age, and the rumen weight to live weight before slaughter and the percentage of total compound stomach weight and the percentage of reticulum to total compound stomach weight were significantly higher in the early supplementation group than in the control group at 200 days of age. From the experimental results, this is due to the fact that early weaning with starter feeds provides more physical stimulation, which is the main reason to promote rumen muscle layer development and rumen weight gain [39]. As rumen function improves, the specific effects of nutritional compensation resulting from this early supplementation at different stages of development are subject to further investigation. The degree of development of the compound stomach of ruminants at a young age affects feed intake and digestive capacity in adulthood, with rumen development being particularly important in determining future production performance. Young ruminants are born with an undeveloped rumen, with no physiological or metabolic functions [40]. With the change in diet, when the liquid feed (breast milk or milk replacer) is changed to solid feed, the digestive tract of young ruminants starts to change, the rumen and reticulum start to grow in volume and weight, the hindgut occupies less, and the microvilli become shorter [41], preparing the way for the change in nutrient absorption and utilization. Kosgey et al. [35] demonstrated that early weaning promotes rumen function. Early supplementation with solid feed is an important driver of rumen epithelial cell development [42]. Previous studies have shown that supplementation of starter feed in the pre-weaning period can significantly increase rumen weight and teat size, enhance rumen function, and ultimately have a positive impact on the health and growth of ruminants [30,43,44,45]. Chai et al. [37] found that the rumen weight of early weaned lambs was not only significantly higher than that of the normal weaning group, but also the percentage of rumen weight to the total compound stomach weight and live weight before slaughter was significantly higher than that of the normal weaning group, indicating that the development rate of the rumen was faster than the overall development rate of the body. 

The small intestine is the main site for nutrient absorption in lambs. The normal development of the small intestine is key to ensuring the absorption and utilization of nutrients in lambs, and ingestion is the main cause of structural and functional changes in the intestine. The weight of the small intestine is closely related to its digestive and absorption capacities of the small intestine. Chai et al. [37] found that the small intestine weight of early weaned lambs at 90 days of age was significantly higher than that of normal-weaned lambs. We found significantly higher jejunal weight in the early supplementation group of lambs than in the control group at 98 days of age, with no significant differences in the data for other intestinal segments. Studies have shown that early supplementation with solid feeds can promote the development of small intestinal mucosa in young ruminants and reduce weaning stress response in young animals by chemically stimulating the regulation of the structure of intestinal microflora [46]. However, it has also been reported that when feed for young ruminants changes from liquid to solid, it is detrimental to the development of the small intestine [47]. At 200 days of age, the percentage of duodenum weight to total gastrointestinal tract weight was significantly lower in the early supplementation group than in the control group, with no significant differences in the data for other intestinal segments. Early supplementation with starter causes the rumen of lambs to develop faster, so the percentage of the small intestine in the weight of the gastrointestinal tract gradually decreases with age, while the percentage of the large intestine in the weight of the gastrointestinal tract remains [17]. In our experiment, there was no significant difference in the length of each intestine or the percentage of their length to the total intestinal length between the two groups, both in the post-weaning and fattening periods. This indicated that early supplementation with starter feed had no effect on the length of the intestine of the lambs.

## 5. Conclusions

Early supplementation with starter improved the growth performance of lambs in the pre-weaning period (7–28 days of age), post-weaning period (28–98 days of age), and slaughter performance at 98 days of age, and promoted rumen development. Early supplementation significantly reduced the ratio of feed-to-weight gain in lambs, with no significant effect on growth performance and slaughter performance during the fattening period. Early supplementation with starters was able to reduce feed costs and improve the performance of lambs, while the growth advantage generated by early supplementation disappeared after 154 days of age.

## Figures and Tables

**Table 1:** Ingredients and nutritional composition of the starter and fattening rations.

Items	Starter Ration (7~98 Days)	Fattening Ration (99~200 Days)
Ingredients, air dry basis, %		
Corn	42.05	39.05
Soybean meal	18.50	11.00
Molasses	5.00	2.60
Whey powder	6.00	0.00
Cotton pulp	4.00	6.00
Puffing of soybean	5.90	2.90
Alfalfa hay	9.20	20.25
Soybean hulls	7.30	16.20
NaCl	0.70	0.70
Stone powder	0.90	0.80
Premix ^1^	0.40	0.50
Flavor agent	0.05	0.00
Nutrient composition ^2^, dry matter basis		
DM, air dry basis	88.49	89.01
ME (MJ/kg)	10.79	9.87
CP (%)	19.16	16.48
Ca (%)	0.66	0.73
P (%)	0.36	0.30
NDF (%)	18.38	18.38
Fat (%)	2.75	2.45
Ash (%)	4.48	4.34
Starch (%)	30.02	28.24

^1^ Contained per kilogram of supplement: 25 mg of Fe, 40 mg of Mn, 40 mg of Zn, 8 mg of Cu, 0.3 mg of I, 0.2 mg of Se, 0.1 mg of Co, 940 IU of vitamin A, 111 IU of vitamin D, 20 IU of vitamin E. ^2^ Dry matter (DM), crude protein (CP), calcium (Ca), phosphorus (P), neutral detergent fiber (NDF), fat, ash and starch were measured values, and metabolic energy (ME) was calculated values.

**Table 2:** Effects of early supplementation on the growth performance of lambs.

Items	Treatment ^4^		SEM	*p*-Value ^5^		
	CON	ES		Treatment	Age	Treatment × Age
ADG ^1^ (g/d)						
Pre-weaning (7–28 days)	36.96 ^b^	52.08 ^a^	3.51	**0.032**	**<0.001**	**0.044**
Post-weaning (29–98 days)	236.35	203.33	6.15	0.400	**<0.001**	**0.031**
Fattening period (99–200 days)	297.09	300.07	7.69	0.556	**<0.001**	0.888
Overall (7–200 days)	221.08	225.31	4.32	0.441	**<0.001**	0.999
ADFI ^2^ (g/d)						
Pre-weaning (7–28 days)	32.26 ^b^	53.76 ^a^	5.00	**0.029**	**<0.001**	0.558
Post-weaning (29–98 days)	639.01 ^b^	657.33 ^a^	18.18	**0.019**	**<0.001**	0.095
Fattening period (99–200 days)	1598.76	1487.78	13.66	0.146	**<0.001**	0.618
Overall (7–200 days)	1152.67 ^b^	1098.10 ^a^	22.45	**0.001**	**<0.001**	0.074
F/G ^3^						
Pre-weaning (7–28 days)	0.27 ^b^	0.59 ^a^	0.04	**0.016**	**<0.001**	0.806
Post-weaning (29–98 days)	1.38	1.41	0.01	0.545	**<0.001**	0.559
Fattening period (99–200 days)	6.36 ^a^	5.86 ^b^	0.11	**0.024**	**<0.001**	0.895

^1^ ADG: average daily gain. ^2^ ADFI: average daily feed intake. ^3^ F/G: ratio of feed to gain. ^4^ Treatment: control group; early supplementation group; *n* = 22. ^5^ Mean values with different superscripts in a treatment differed significantly (*p* < 0.05). The significance of bold values as treatment differed significantly.

**Table 3:** Effect of early supplementation on body size in lambs.

Items	Age/d	Treatment ^1^		SEM	*p*-Value ^2^
		CON	ES		
Body height	7	40.25	41.42	0.78	0.387
	28	45.33 ^a^	45.08 ^b^	0.29	**0.045**
	56	49.27 ^b^	50.45 ^a^	0.37	**0.044**
	84	54.70	55.20	0.37	0.212
	112	61.00 ^b^	61.43 ^a^	0.28	**0.030**
	140	65.29	66.00	0.39	0.145
	168	68.00	69.13	0.44	0.796
	196	74.00	74.40	0.40	0.296
Body length	7	35.08	35.42	0.23	0.639
	28	36.36 ^b^	36.45 ^a^	0.33	**0.042**
	56	45.13	46.88	0.41	0.573
	84	49.75	53.13	0.65	0.287
	112	57.22	59.33	0.86	0.171
	140	62.78 ^b^	64.00 ^a^	0.51	**0.041**
	168	69.11	69.78	0.62	0.879
	196	73.78	76.44	0.66	0.447
Chest circumference	7	40.08	40.67	0.03	0.860
	28	39.63	41.25	0.35	0.219
	56	47.89	50.56	0.52	0.770
	84	56.88	59.38	0.52	0.769
	112	63.13	66.75	0.60	0.319
	140	73.44	73.22	0.38	0.139
	168	79.44	81.11	0.72	0.649
	196	83.56	85.11	0.74	0.861
Tube circumference	7	5.75	5.21	0.58	0.108
	28	5.13	5.17	0.56	0.347
	56	5.75	5.88	0.07	0.522
	84	6.08	5.96	0.06	0.153
	112	6.69 ^b^	6.94 ^a^	0.08	**0.016**
	140	7.58	7.41	0.07	0.100
	168	8.10	7.95	0.07	0.512
	196	8.15	8.10	0.50	0.334

^1^ Treatment: control group; early supplementation group; *n* = 22. ^2^ Mean values with different superscripts in a treatment differed significantly (*p* < 0.05). The significance of bold values as treatment different significantly.

**Table 4:** Effect of early supplementation on faecal dry matter and fibre digestibility in *Hu* lambs.

Items	Treatment ^4^		SEM	*p*-Value ^5^
	CON	ES		
DM ^1^				
50–70 days	94.99	95.81	0.23	0.077
91–112 days	96.63	96.37	0.24	0.618
133–154 days	93.46 ^b^	95.89 ^a^	1.64	**0.012**
155–175 days	83.13 ^b^	97.40 ^a^	2.65	**0.001**
176–200 days	97.70	96.52	0.34	0.094
NDF ^2^				
50–70 days	42.37	46.29	1.19	0.102
91–112 days	42.21	47.03	1.29	0.063
133–154 days	52.14	53.94	0.82	0.299
155–175 days	48.11 ^b^	54.58 ^a^	1.54	**0.018**
176–200 days	50.45 ^b^	54.70 ^a^	0.93	**0.003**
ADF ^3^				
50–70 days	25.12	24.99	0.99	0.951
91–112 days	26.67	26.73	0.67	0.969
133–154 days	34.94	37.40	0.72	0.088
155–175 days	35.08 ^b^	39.61 ^a^	1.16	**0.041**
176–200 days	36.01 ^b^	38.97 ^a^	0.70	**0.005**

^1^ DM: dry matter; ^2^ NDF: neutral detergent fiber; ^3^ ADF: acid detergent fiber. ^4^ Treatment: control group; early supplementation group; *n* = 22. ^5^ Mean values with different superscripts in a treatment differed significantly (*p* < 0.05). The significance of bold values as treatment differed significantly.

**Table 5:** Effect of early supplementation on slaughter performance in lambs.

Items	Treatment ^1^		SEM	*p*-Value ^2^
	CON	ES		
98 days of age				
Live weight before slaughter (kg)	20.94 ^b^	25.56 ^a^	0.90	**0.004**
Carcass weight (kg)	10.04 ^b^	12.26 ^a^	0.51	**0.024**
Dressing percentage (%)	47.67	47.97	0.75	0.853
200 days of age				
Live weight before slaughter (kg)	45.10	46.57	1.09	0.114
Carcass weight (kg)	23.87	23.38	0.56	0.071
Dressing percentage (%)	52.39	52.04	0.77	0.827

^1^ Treatment: control group; early supplementation group; *n* = 8. ^2^ Mean values with different superscripts in a treatment differed significantly (*p* < 0.05). The significance of bold values as treatment differed significantly.

**Table 6:** Effect of early supplementation on the gastrointestinal indices in 90- and 200-day-old lambs.

Items		Treatment ^4^		SEM	*p*-Value ^5^
		CON	ES		
98 days of age					
Rumen	Weight (g)	610.00 ^b^	810.00 ^a^	50.10	**0.035**
	Percentage of LWBS ^1^ (%)	2.74	3.48	0.22	0.085
	Percentage of TCSW ^2^ (%)	73.48	74.04	1.05	0.805
Reticulum	Weight (g)	80.00 ^b^	108.00 ^a^	6.48	**0.018**
	Percentage of LWBS (%)	0.37	0.46	0.03	0.124
	Percentage of TCSW (%)	8.35 ^b^	9.98 ^a^	1.19	**0.037**
Omasum	Weight (g)	58.57	52.86	4.02	0.499
	Percentage of LWBS (%)	0.25	0.26	0.02	0.904
	Percentage of TCSW (%)	5.17	5.89	0.31	0.266
Abomasum	Weight (g)	115.71	98.57	6.67	0.211
	Percentage of LWBS (%)	0.45	0.48	0.03	0.656
	Percentage of TCSW (%)	11.76	11.10	0.89	0.728
Duodenum	Weight (g)	30.00	40.00	4.47	0.284
	Percentage of LWBS (%)	0.14	0.15	0.02	0.771
	Percentage of TGIW ^3^ (%)	1.66	2.25	0.23	0.243
Jejunum	Weight (g)	607.14 ^b^	726.67 ^a^	29.36	**0.035**
	Percentage of LWBS (%)	2.85	2.92	0.11	0.759
	Percentage of TGIW (%)	33.05	33.20	1.15	0.954
Ileum	Weight (g)	17.14	20.00	2.22	0.545
	Percentage of LWBS (%)	0.08	0.08	0.01	0.924
	Percentage of TGIW (%)	0.96	1.10	0.14	0.643
Cecum	Weight (g)	35.71	46.00	3.02	0.092
	Percentage of LWBS (%)	0.15	0.18	0.02	0.570
	Percentage of TGIW (%)	1.96	2.44	0.15	0.140
Colon and Rectum	Weight (g)	241.43	262.00	5.90	0.085
	Percentage of LWBS (%)	1.03	1.17	0.04	0.104
	Percentage of TGIW (%)	12.47	13.23	0.37	0.345
200 days of age					
Rumen	Weight (g)	700.00	755.00	29.57	0.377
	Percentage of LWBS (%)	1.52 ^b^	1.72 ^a^	0.04	**0.003**
	Percentage of TCSW (%)	66.69 ^b^	70.27 ^a^	0.90	**0.041**
Reticulum	Weight (g)	115.00	118.33	4.32	0.719
	Percentage of LWBS (%)	0.25	0.27	0.01	0.068
	Percentage of TCSW (%)	10.47 ^b^	11.47 ^a^	0.25	**0.039**
Omasum	Weight (g)	93.75	88.75	3.75	0.524
	Percentage of LWBS (%)	0.20	0.19	0.12	0.471
	Percentage of TCSW (%)	8.95	8.37	0.40	0.479
Abomasum	Weight (g)	144.17	123.85	5.62	0.070
	Percentage of LWBS (%)	0.31	0.27	0.01	0.161
	Percentage of TCSW (%)	12.53	12.21	0.46	0.733
Duodenum	Weight (g)	30.00	28.57	1.95	0.730
	Percentage of LWBS (%)	0.07	0.05	0.01	0.123
	Percentage of TGIW3 (%)	1.62 ^a^	1.14 ^b^	0.11	**0.022**
Jejunum	Weight (g)	651.43	700.00	24.01	0.331
	Percentage of LWBS (%)	1.56	1.42	0.05	0.211
	Percentage of TGIW (%)	31.62	31.52	0.63	0.938
Ileum	Weight (g)	21.43	24.29	1.63	0.403
	Percentage of LWBS (%)	0.05	0.05	0.00	0.798
	Percentage of TGIW (%)	0.96	1.09	0.06	0.291
Cecum	Weight (g)	51.67	46.92	2.23	0.298
	Percentage of LWBS (%)	0.11	0.10	0.00	0.678
	Percentage of TGIW (%)	2.17	2.27	0.09	0.596
Colon and Rectum	Weight (g)	350.83	320.77	13.44	0.273
	Percentage of LWBS (%)	0.74	0.72	0.03	0.759
	Percentage of TGIW (%)	14.46	15.65	0.49	0.230

^1^ LWBS: live weight before slaughter. ^2^ TCSW: total compound stomach weight. ^3^ TGIW: total gastrointestinal weight. ^4^ Treatment: control group; early supplementation group; *n* = 8. ^5^ Mean values with different superscripts in a treatment differed significantly (*p* < 0.05). The significance of bold values as treatment differed significantly.

**Table 7:** Effect of early supplementation on the intestinal relative length of *Hu* lambs.

Items		Treatment ^1^		SEM	*p*-Value
		CON	ES		
98 days of age					
Duodenum	Length (cm)	70.00	83.93	5.73	0.289
	Length to total intestinal tract (%)	4.22	3.53	0.38	0.386
Jejunum	Length (cm)	2233.50	2298.71	56.58	0.588
	Length to total intestinal tract (%)	77.74	76.65	1.78	0.775
Ileum	Length (cm)	31.20	39.14	4.09	0.315
	Length to total intestinal tract (%)	1.05	1.31	0.14	0.365
Cecum	Length (cm)	30.03	25.90	1.86	0.259
	Length to total intestinal tract (%)	0.86	0.86	0.09	0.982
Colon and Rectum	Length (cm)	473.16	528.00	45.92	0.575
	Length to total intestinal tract (%)	16.11	17.63	1.50	0.635
200 days of age					
Duodenum	Length (cm)	61.68	60.23	2.80	0.802
	Length to total intestinal tract (%)	1.57	1.71	0.10	0.507
Jejunum	Length (cm)	2753.37	2669.52	45.93	0.370
	Length to total intestinal tract (%)	76.69	75.35	0.54	0.226
Ileum	Length (cm)	52.79	53.52	2.83	0.900
	Length to total intestinal tract (%)	1.47	1.52	0.09	0.775
Cecum	Length (cm)	32.39	30.92	1.07	0.355
	Length to total intestinal tract (%)	0.91	0.87	0.03	0.509
Colon and Rectum	Length (cm)	691.87	724.69	17.57	0.362
	Length to total intestinal tract (%)	19.33	20.52	0.53	0.274

^1^ Treatment: control group; early supplementation group; *n* = 8.

## Data Availability

Data will be stored with Jianfeng Xu and Ting Liu.

## References

[1] Knights M., Siew N., Ramgattie R., Singh-Knights D., Bourne G. (2012). Effect of time of weaning on the reproductive performance of Barbados Blackbelly ewes and lamb growth reared in the tropics. Small Rumin. Res..

[2] Zhong R.Z., Sun H.X., Li G.D., Liu H.W., Zhou D.W. (2014). Effects of inoculation with rumen fluid on nutrient digestibility, growth performance and rumen fermentation of early weaned lambs. Livest. Sci..

[3] Sena H., Santos K., Silva M., McMannus C., Ramos A., Bernal F. (2013). Learning, memory and stress evaluation in lambs weaned at different ages. J. Anim. Sci. Adv..

[4] Napolitano F., Rosa G.D., Sevi A. (2008). Welfare implications of artificial rearing and early weaning in sheep. Appl. Anim. Behav. Sci..

[5] Blanco M., Ripoll G., Albertí P., Sanz A., Revilla R., Villalba D., Casasús I. (2008). Effect of early weaning on performance, carcass and meat quality of spring-born bull calves raised in dry mountain areas. Livest. Sci..

[6] Sefidbakht N., Farid A. (1977). Effect of early weaning and hormone treatments on induction of estrus, conception and lambing of fall-lambing Karakul ewes. J. Anim. Sci..

[7] Budzynska M., Weary D.M. (2008). Weaning distress in dairy calves: Effects of alternative weaning procedures. Appl. Anim. Behav. Sci..

[8] De Passillé A., Borderas T., Rushen J. (2011). Weaning age of calves fed a high milk allowance by automated feeders: Effects on feed, water, and energy intake, behavioral signs of hunger, and weight gains. J. Dairy Sci..

[9] Khan M., Lee H., Lee W., Kim H., Kim S., Ki K., Ha J., Lee H., Choi Y. (2007). Pre-and postweaning performance of Holstein female calves fed milk through step-down and conventional methods. J. Dairy Sci..

[10] Enríquez D., Hötzel M.J., Ungerfeld R. (2011). Minimising the stress of weaning of beef calves: A review. Acta Vet. Scand..

[11] Vi R., Mcleod K., Klotz J.L., Heitmann R.N. (2004). Rumen Development, Intestinal Growth and Hepatic Metabolism in the Pre- and Postweaning Ruminant. J. Dairy Sci..

[12] Drackley J.K. (2008). Calf nutrition from birth to breeding. Vet. Clin. N. Am. Food Anim. Pract..

[13] Yang B., He B., Wang S., Liu J., Wang J. (2015). Early supplementation of starter pellets with alfalfa improves the performance of pre-and postweaning Hu lambs. J. Anim. Sci..

[14] Reddy K.E., Jeong J., Baek Y.-C., Oh Y.K., Kim M., So K.M., Kim M.J., Kim D.W., Park S.K., Lee H.-J. (2017). Early weaning of calves after different dietary regimens affects later rumen development, growth, and carcass traits in Hanwoo cattle. Asian-Australas. J. Anim. Sci..

[15] Calder P.C., Krauss-Etschmann S., de Jong E.C., Dupont C., Frick J.-S., Frokiaer H., Heinrich J., Garn H., Koletzko S., Lack G. (2006). Early nutrition and immunity–progress and perspectives. Br. J. Nutr..

[16] Bach A., Giménez A., Juaristi J., Ahedo J. (2007). Effects of physical form of a starter for dairy replacement calves on feed intake and performance. J. Dairy Sci..

[17] Zhiyuan M., Fei L., Fadi L., Chong L., Weiming W., Defu T. (2015). Effects of early weaning on body size, slaughter performance and visceral development of lake sheep. J. Domest. Anim. Ecol..

[18] (2022). Standard for Feeding Meat Sheep in the Agricultural Industry of the People’s Republic of China.

[19] AOAC (2006). Association of Official Analytical Chemists International. Official Methods of Analysis of AOAC International.

[20] Van Soest P.J., Robertson J.B., Lewis B.A. (1991). Methods for dietary fiber, neutral detergent fiber, and nonstarch polysaccharides in relation to animal nutrition. J. Dairy Sci..

[21] Raineri C., Stivari T.S.S., Gameiro A.H. (2015). Lamb Production Costs: Analyses of Composition and Elasticities Analysis of Lamb Production Costs. Asian-Australas. J. Anim. Sci..

[22] Lucas A. (1991). Programming by early nutrition in man. Ciba Found Symp..

[23] Shaobo Y., Bing Y., Shanshan W., Jiakun W. (2020). Microbial succession in the digestive tract of young ruminants and regulation of digestive tract development. Feed. Ind..

[24] Emsen E., Yaprak M., Bilgin O.C., Emsen B., Ockerman H.W. (2004). Growth performance of Awassi lambs fed calf milk replacer. Small Rumin. Res..

[25] Wardrop I., Coombe J. (1961). The development of rumen function in the lamb. Aust. J. Agric. Res..

[26] Castells L., Bach A., Aris A., Terré M. (2013). Effects of forage provision to young calves on rumen fermentation and development of the gastrointestinal tract. J. Dairy Sci..

[27] Castells L., Bach A., Araujo G., Montoro C., Terré M. (2012). Effect of different forage sources on performance and feeding behavior of Holstein calves. J. Dairy Sci..

[28] Terre M., Pedrals E., Dalmau A., Bach A. (2013). What do preweaned and weaned calves need in the diet: A high fiber content or a forage source?. J. Dairy Sci..

[29] Wenqin H., YM Z., Fan G., Yubin Z., Kai C., Shiqin W., Qiyu D., Naifeng Z. (2020). Sustained effects of early weaning and supplementation with milk replacer on growth performance, digestive performance, serum biochemical parameters and meat quality of lambs. J. Anim. Nutr..

[30] Pazoki A., Ghorbani G., Kargar S., Sadeghi-Sefidmazgi A., Drackley J., Ghaffari M. (2017). Growth performance, nutrient digestibility, ruminal fermentation, and rumen development of calves during transition from liquid to solid feed: Effects of physical form of starter feed and forage provision. Anim. Feed. Sci. Technol..

[31] Nejad J.G., Torbatinejad N., Naserian A.A., Kumar S., Kim J., Song Y., Ra C., Sung K. (2012). Effects of processing of starter diets on performance, nutrient digestibility, rumen biochemical parameters and body measurements of Brown Swiss dairy calves. Asian-Australas. J. Anim. Sci..

[32] Kumar R.S., Ramesh V., Kumar K.S., Thiruvenkadan A. (2017). Early weaning and concentrate supplementation on body condition score of Mecehri ewes and lambs. Indian J. Anim. Prod. Manag..

[33] Bosi P., Gremokolini C., Trevisi P. (2003). Dietary Regulations of the Intestinal Barrier Function at Weaning. Asian Australas. J. Anim. Sci..

[34] Norouzian M.A., Valizadeh R., Vahmani P. (2011). Rumen development and growth of Balouchi lambs offered alfalfa hay pre-and post-weaning. Trop. Anim. Health Prod..

[35] Kosgey I., Baker R., Udo H., Van Arendonk J.A. (2006). Successes and failures of small ruminant breeding programmes in the tropics: A review. Small Rumin. Res..

[36] Dianfen L. (2010). Effect of Milk Replacer Composition on Lamb Growth and Early Weaning on Ewe Reproductive Function. Master’s Thesis.

[37] Jianmin C., Qiyu D., Yan T., Haichao W., Naifeng Z. (2014). Effect of early weaning time on tissue and organ development, slaughter performance and meat quality of lambs in Hu sheep. J. Anim. Nutr..

[38] Ramos Z., De Barbieri I., van Lier E., Montossi F. (2020). Carcass and meat quality traits of grazing lambs are affected by supplementation during early post-weaning. Small Rumin. Res..

[39] Amaral C., Sugohara A., Resende K.T., Machado M., Cruz C. (2005). Performance and ruminal morphologic characteristics of Saanen kids fed ground, pelleted or extruded total ration. Small Rumin. Res..

[40] Warnes D.M., Seamark R.F., Ballard F. (1977). The appearance of gluconeogenesis at birth in sheep. Activation of the pathway associated with blood oxygenation. Biochem. J..

[41] Yang B., Le J., Wu P., Liu J., Guan L.L., Wang J. (2018). Alfalfa Intervention Alters Rumen Microbial Community Development in Hu Lambs During Early Life. Front. Microbiol..

[42] Lv X., Chai J., Diao Q., Huang W., Zhuang Y., Zhang N. (2019). The signature microbiota drive rumen function shifts in goat kids introduced to solid diet regimes. Microorganisms.

[43] Xixin Y. (2011). Effect of Protein Level and Feeding amount on Growth Performance and Digestive Metabolism of Early Weaned Lambs. Master’s Thesis.

[44] Sun D., Mao S., Zhu W., Liu J. (2018). Effect of starter diet supplementation on rumen epithelial morphology and expression of genes involved in cell proliferation and metabolism in pre-weaned lambs. Animal.

[45] Berends H., Van Reenen C., Stockhofe-Zurwieden N., Gerrits W. (2012). Effects of early rumen development and solid feed composition on growth performance and abomasal health in veal calves. J. Dairy Sci..

[46] Dsab C., Smab C., Wzab C., Jlab C. (2019). Effects of starter feeding on caecal mucosal bacterial composition and expression of genes involved in immune and tight junctions in pre-weaned twin lambs. Anaerobe.

[47] Shanshan W. (2015). Effect of Two Early Weaning Supplementation Patterns on the Development of Intestinal Barrier Function in Lambs. Master’s Thesis.

